# Molecular Evaluation of t(14;18)(bcl-2/IgH) Translocation in Follicular Lymphoma at Diagnosis Using Paraffin-Embedded Tissue Sections

**DOI:** 10.5505/tjh.2012.93898

**Published:** 2012-06-15

**Authors:** Nur Selvi, Buket Kosova, Mine Hekimgil, Cumhur Gündüz, Burçin Tezcanlı Kaymaz, Emin Karaca, Güray Saydam, Murat Tombuloğlu, Filiz Büyükkeçeci, Seçkin Çağırgan, Yeşim Ertan, Nejat Topçuoğlu

**Affiliations:** 1 Ege University, School of Medicine, Medical Biology Department, İzmir, Turkey; 2 Ege University, School of Medicine, Hematology Department, İzmir, Turkey; 3 Ege University, School of Medicine, Pathology Department, İzmir, Turkey; 4 Ege University, School of Medicine, Medical Genetics Department, İzmir, Turkey

**Keywords:** Follicular lymphoma, FISH, Multiplex PCR, Semi-nested PCR

## Abstract

**Objective:** Follicular lymphoma (FL) is one of the most common lymphomas, and is characterized by t(14;18)(q32;q21) in more than 80% of patients. The aim of this study was to determine the rate of t(14;18) positivity based onthe detection of mbr or mcr in paraffin-embedded tissue samples.

**Material and Methods:** The study included 32 paraffin-embedded tissue samples collected from 32 consecutive FL patients that were diagnosed and followed-up at our hospital between 1999 and 2006. The MBR breakpoint wasidentified based on real-time PCR using a LightCycler v.2.0 t(14;18) Quantification Kit (MBR), multiplex PCR, and seminestedPCR. To identify the mcr breakpoint, real-time PCR was performed using specific primers and the FastStart DNAMaster SYBR Green I Kit. To detect t(14;18) via fluorescence in situ hybridization (FISH) nuclei from paraffin-embeddedtissue sections were extracted and used together with LSI IgH (immunoglobulin heavy chain) (spectrum green)/bcl-2(B-cell leukemia-lymphoma 2) (spectrum orange) probes.

**Results:** The DNA and nuclei isolation success rate for B5 formalin-fixed, paraffin-embedded tissue sections (n = 12)was 42% and 33%, respectively, versus 95% and 60%, respectively, for 20 tissue sections fixed in formalin only. In all,24 paraffin-embedded tissue sections were analyzed and mbr positivity was observed in the DNA of 82.14% via seminested PCR, in 53.57% via multiplex PCR, and in 28.57% via real-time PCR. We did not detect mcr rearrangementin any of the samples. In all, 15 of 16 patients (93.75%) whose nuclei were successfully isolated were observed to bet(14;18) positive via the FISH method.

**Conclusion:** Semi-nested PCR and FISH facilitated the genetic characterization of FL tumors. As such, FISH and PCR complement each other and are both essential for detecting t(14;18) translocation.

## Abstract (tr)

**Amaç:** Foliküler lenfoma, lenfomalarda sıklıkla görülür ve hastaların %80 inden fazlası t(14;18) (q32;q21) translokasyonu ile karekterizedir. Bu çalışmada; 32 adet parafine gömülü doku örneğinde t(14;18) translokasyonunun tespiti için MBR ve mcr pozitifliği değerlendirildi.

**Gereç ve Yöntemler:** MBR kırılma noktası LightCycler t(14;18) Quantification Kit (MBR) ile gerçek zamanlı PCR, multiplex PCR, and semi-nested PCR yöntemleri kullanılarak araştırıldı. mcr kırılma bölgesi spesifik primerler kullanılarak; Fast Start DNA Master SYBR Green I kiti kullanılarak gerçek-zamanlı bir cihazı olan LightCycler’ da değerlendirildi. t(14;18) (q32;q21) translokasyonunun floresans in situ hibridizasyon (FISH) yöntemi ile tespit edilebilmesi için; parafine-gömülü doku örneklerinden nükleus izolasyonu yapıldı ve örnekler IgH/bcl-2 dual color,dual fusion translokasyon probu ile inkübe edildi.

**Bulgular:** B5- Formalin ile fikse edilen parafine-gömülü 12 adet doku örneğinde DNA ve nükleus izolasyonununda sırasıyla % 42 ve % 33 başarı elde edilirken, formalinle fikse edilen 20 adet doku örneğinde bu başarı sırasıyla % 95 ve % 60 e kadar yükselmiştir. DNA izolasyonu başarılı olan 24 adet parafine-gömülü doku örneğinde; MBR pozitifliği % 82.14 başarı gösterirken, multipleks PCR sonuçlarına göre başarı oranı % 53.57 ve gerçek-zamanlı PCR ile % 28.57 olarak bulunmuştur.MBR negatif örneklerde mcr yeniden düzenlenmesi çalışılmış pozitif sonuç elde edilmemiştir. Nukleus izolasyonu başarılı olan 16 örneğin 15 inde (% 93.75) t(14;18) (q32;21) translokasyonu FISH yöntemi ile pozitif olarak bulunmuştur.

**Sonuç:** Sonuç olarak semi nested PCR ve FISH yöntemleri tümörün genetik karekterizasyonunun aydınlatılması için kullanılabilecek iki fonksiyonel moleküler metoddur. Bu nedenle FISH ve PCR yöntemlerinin birbirini tamamladığı ve t(14;18) translokasyonunun tespitinde oldukça önemli birer parametre olduğu düşünülmektedir.

## INTRODUCTION

The t(14;18) (q32;q21) chromosomal translocation is observed in approximately 80% of all follicular lymphoma (FL) patients. FL, a sub-type of non-Hodgkin’s lymphoma (NHL), occurs in about 13% of the Indian population, versus 40% of western populations. This translocation juxtaposes the immunoglobulin heavy chain enhancer region (IgH Eμ) at 14q32 with the bcl-2 (B-cell leukemialymphoma 2) oncogene at 18q21 [1]. As a result of this translocation, there is an overexpression of the bcl-2 gene, which codes for the bcl-2 protein [2]. This protein blocks apoptosis and cell death, and its overexpression is considered an important factor related to multiple drug resistance and lack of response to chemotherapy [3]. The IgH/bcl-2 rearrangement can be detected via southern blotting—a laborious procedure—or by polymerase chain reaction (PCR), which is a very sensitive technique, especially for the detection of minimal residual disease (MRD) [4]. 

PCR can detect 1 circulating lymphoid cell carrying the t(14;18) translocation from among 1 x 105-1 x 106 normal cells, making it possible to detect MRD in the bone marrow or peripheral blood of FL patients [5] as a means of monitoring their response to chemotherapy (associated or not to monoclonal antibody anti-CD-20), and to allogeneic or autologous stem cell transplantation [6]. At the molecular level, breakpoints on chromosome 14 occur within the joining region genes (JH) of the IgH locus. Most breakpoints on chromosome 18 are clustered within a 150-base pair (bp) segment at the 3’ untranslated end of exon 3 of bcl-2, designated as the major breakpoint region (MBR) [7]. Additional breakpoints are described in 30 kb 3’ of the MBR in the minor cluster region (mcr). A third breakpoint cluster region—the 5'-bcl-2 or variant cluster region—exists on 5' of the bcl-2 gene [8]. 

Detection of IgH/bcl-2 fusion in FL is clinically important for establishing a diagnosis and monitoring disease progression. The aim of the present study was to compare the efficacy of PCR and fluorescence in situ hybridization (FISH) in detecting the t(14;18) (q32;q21)-IgH/bcl-2 rearrangement in paraffin-embedded tissue samples.

## MATERIALS AND METHODS

The study included 32 paraffin-embedded tissue samples collected from 32 consecutive FL patients that were diagnosed and followed-up at our hospital between 1999 and 2006. The FL group included 19 female (55.88%) and 13 male (44.12%) FL patient with a mean age of 58±06 years (range: 32-78 years). The control group included paraffin-embedded tissue samples obtained from the tonsils of 17 healthy volunteers (mean age: 42.35±24.75 years).

**DNA extraction from paraffin-embedded tissues **

Sections 25-50 μm thick were obtained from paraffin block samples. Following xylene treatment at 60 °C for 15 min, pellets were incubated for 10 min in a 50% xylene-50% ethanol mixture, and then in 100%, 70%, and 50% ethanol serials. Then, 200 μL of digestion buffer and 40 μL of proteinase K were added to the tissue pellets, followed by overnight incubation at 37 °C. Next, the pellets were incubated for 2 h at 55 °C with 20 μL of proteinase K. Following incubation for 10 min at 70 °C with 200 μL of binding buffer, the mixture was transferred into collection tubes. With the addition of isopropanol the protein contents were eliminated from the nucleic acids. Following 2 washing steps, genomic DNA was dissolved in 100 μL of elution buffer that was warmed to 70 °C in advance. A NanoDrop spectrophotometer was used to measure DNA concentrations ranging from 12.4 to 441 ng μL–1. 

**LightCycler t(14;18) Quantification Kit (MBR) **

A 200-bp fragment of the t(14;18) translocation resulting from MBR breakpoints was amplified via PCR using specific primers. The amplicon was detected via fluorescence using a specific pair of hybridization probes that consisted of 2 different short oligonucleotides that hybridize to an internal sequence of the amplified fragment during the annealing phase of the amplification cycle. One probe was labeled at the 5’ end with Light Cycler RED 640, and to avoid extension, was modified at the 3’-end by phosphorylation. The other probe was labeled at the 3’ end with fluoresce in. Only after hybridization to the template DNA did the 2 probes come into close proximity, resulting in fluorescence resonance energy transfer (FRET) between the 2 fluorophores. During FRET, fluoresce in— the donor fluorophore—was excited by the Light Cycler’s light source, and some of the excitation energy was transferred to Light Cycler Red 640, and then measured via the Light Cycler instrument. Tissue plasminogen activator (tPA) was processed as a reference gene. The kit utilized the “hot start” method using Fast Start Taq polymerase, which facilitates detection of 1 t(14;18) translocation positive cell from among 5x104 -1x105 peripheral blood mononuclear cells in less then 60 min. 

MBR rearrangement analysis using multiplex PCR 

All 26 DNA samples were tested for the IgH/bcl-2 rearrangement using a multiplex PCR protocol, and specific primers for the MBR and jH consensus regions (Table 1), as previously described [21]. Primer sequences and PCR conditions were based on previously described protocols optimized for genomic DNA, and were nearly identical to those used in recent studies [13,14]. PCR products were obtained in positive cases via MBR determinations ranging from approximately 100-275 bp. In addition, PCR sensitivity was documented by running positive control DNA samples (MBR control, Roche Diagnostics, Germany). 

**Figure 1:**
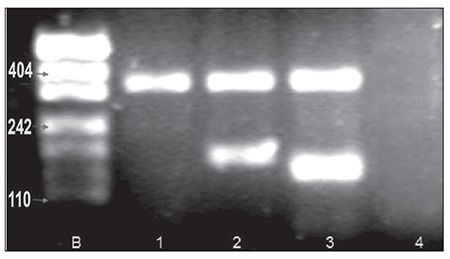
Representative gel photo of multiplex PCR results. Minor band shows PCR amplification products (120-200 bp). The band above shows the multiplex PCR strategy in which control primers were used to detect IgH/bcl-2 rearrangement. The first line represents the 50-bp DNA ladder [PUC 19 DNA/MspI (HpaII)]. Interpretation is as follows: Sample 1: MBR-negative case; samples 2 and 3: MBR-positive cases; sample 4: negative control.

In the present study paraffin-extracted DNA from lymphoid tissue samples fixed in formalin and B5-formalin were evaluated for MBR rearrangement via multiplex PCR. The MBR determinations were analyzed in duplicate, with the first PCR run containing only the specific primers, and the second PCR run containing a multiplex of specific primers and control primers for the β-globulin gene. The β-globulin gene control product (350 bp) was used in this assay to determine the quality of the DNA and PCR conditions. The 25-μL PCR reactions contained the following reagents; 1x PCR buffer, 200 μmol L–1 of deoxynucleoside triphosphate (dNTP), 15 pmol MBR primer, 15 pmol jH primer, 2 pmol control primers, 1.25 U Taq gold, 2.0 mmol L–1 of MgCl2, and 100 ng of paraffin-extracted DNA. Amplification conditions for the MBR reactions were 10 min at 95 °C (hot start), 35 cycles at 94 °C for 1 min, 60 °C for 1 min, and 72 °C for 1 min; the final step was final extension at 72 °C for 10 min, followed by hold at 4 °C. All PCR reactions were performed in a Genius thermocycler. Figure 1 shows a representative PCR gel image.

**Detection of MBR rearrangement in paraffinembeddedtissue (semi-nested PCR)**

**Figure 2:**
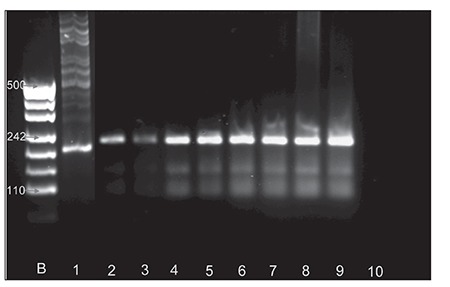
Representative gel photo of semi-nested PCR results. The first line on the left represents the 50-bp DNA ladder (Fermentas). Sample 1: MBR-positive control (Roche Diagnostic); samples 2-9: MBR-positive samples (each sample shows the PCR amplification products [50 bp] using primers specific to the bcl-2 MBR and JH consensus; sample 10: negative control.

Paraffin-embedded tissue samples were analyzed for the presence of MBR rearrangement via different semi-nested PCR reactions. Each reaction utilized sense primers. Wehave combined the techniques which are described in twostudies by Gisele et al and Einerson et al [2,21]. The listof used primers is given in Table 2. PCR reactions wereconducted in duplicate in a Genius thermocycler. First weused multiplex PCR reactions and the second PCR reactioncontained 2.5 μL of the first PCR product for each 25-μLreaction, 1x Taq polymerase buffer, 200 μmol dNTP, 15pmol internal MBR and external jH[2], and 1.5 U Taqpolymerase, with denaturation at 94 °C for 45 s, annealingat 66 °C for 45 s, extension at 72 °C for 30 s, and a finalextension at 72 °C for 5 min. PCR results are shown in Figure 2.

**Detection of mcr rearrangement**

The Light Cycler Fast Start DNA Master SYBR Green IKit was used to detect mcr rearrangement. In combination with the Light Cycler system and suitable primers, thekit facilitates highly sensitive detection and quantification of defined DNA sequences. The present study used mcrforward and Jh reverse primers [2], and modified kit procedureswere employed. The list of primers is shown in Table 1.

PCR was performed according to the SYBR Green I Kitprotocol. PCR products were visualized under UV light on2% agarose gel and stained with ethidium bromide. Themcr rearrangement product ranged from 600 to 700 bp.The positive control used for the mcr rearrangement wasproduced by InVivoScribe (Carlsbad, Ca, USA).

**Detection of t(14;18) translocation via FISH**

FISH is difficult to perform using thin sections of paraffin-embedded lymphoid tissues because of high cellularityand truncated cells interfere with accurate scoring of individualnuclei. We used Peterson et al.’s method [15] andan Insitus Biotechnologies kit for nuclei isolation; for this purpose 25-50-μ thick paraffin-embedded tissue sampleswere collected into microcentrifuge tubes.

**Extraction of the nuclei**

Paraffin was dissolved in xylene at room temperaturefor 30 min, at 60 °C with two 10-min changes of xylene(200 μL each) in the microcentrifuge tubes. Each tissuepellet was dehydrated in 100% and then in 50% ethanolfor 5 min each. Enzymatic digestion was then performedwith proteinase K (0.005%) in a collagenase solution. Thespecimen was incubated at 37 °C for overnight.

Nuclei were pelleted using microcentrifuge tubes at6000 rpm for 5 min. For high-quality nuclei isolation, weused the Insitus Biotechnologies kit, which was designedfor paraffin-embedded tissue slides and bifunctional skipdewax. Nuclei were resuspended in 200 μL of fixative (3 parts methanol and 1 part glacial acetic acid). Nuclei suspensions were stored at –20 °C.

**FISH probe and slide preparation**

**Figure 3:**
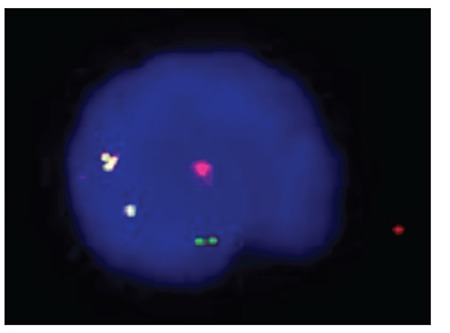
FISH analysis of an abnormal nucleus from FL patient 10. The interphase FISH shows a 1R1G2F signal pattern. R: Red bcl-2 signals; G: green IgH signals; F: IgH/bcl-2 fusion signals.

**Figure 4:**
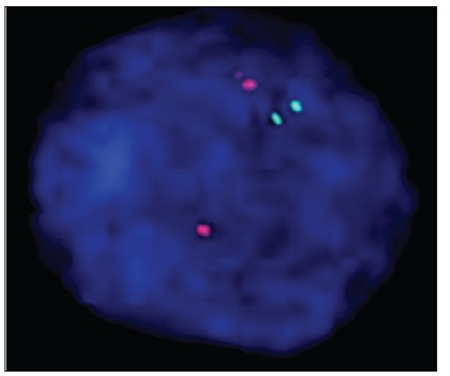
FISH analysis of an abnormal nucleus from FL patient 17. The interphase FISH shows a 2R2G signal pattern. R: Red bcl-2 signals; G: green IgH signals.

One drop of fixed nuclei suspension was placed onslides and air-dried. Slides were incubated at 37 °C in 2xSSC solution for 30 min. Slides were then washed twicein bidistilled water, and then dehydrated with 70%, 85%,and 100% ethanol for 2 min each. Probe mixture (7 μLof hybridization buffer/1 μL of probe [IgH/bcl2 dualt(14;18)]/2 μL of purified water) was added to each slide and a 22 x 22-mm cover slip was placed over each hybridizationsite and sealed with rubber cement. The slides and probe were co-denatured in a Vysis Hybrite set at a melting temperature of 73 °C and melting time of 5 min, followed by hybridization at 37 °C for 16-20 h. Cover slips were removed and slideswere washed for 5 min in 0.4x SSC/NP40 at 73 °C andtransferred to 2x SSC/NP40 at room temperature for 30 s.Nuclei were counterstained with 5 μL of 40-60-diamidine-2-phenylindole dihydrochloride (DAPI). A Vysis LSI IgH/bcl-2 dual color, dual fusion t(14;18) probe was used for FISH analysis of all nuclei. Cells were visualized with afluorescent microscope (Olympus BX50) and 1000 cells were counted in each slide. Representative cells were photographedusing a computer-based imaging system. Figures 3 and 4 show a positive and negative result for bcl2/IgH rearrangement via FISH, respectively.

## RESULTS

A comparison of the PCR and FISH results is shown in Table 3. In total, 26 (81.25%) of the 32 patients with MBR rearrangement were identified via FISH and/or PCR molecular methods. DNA isolation was successfully performed with 24 (24/32) paraffin-embedded tissue samples, whereas among the 8 samples from which DNA was not successfully isolated 7 were fixed with B5-formalin and 1 was fixed with formalin. In all, PCR failed with 8 (25%) of 32 FL patients’ samples (no ß-globulin products were obtained); of the 24 samples in which PCR was successful, 19 (79%) were determined to be positive via semi-nested PCR, 10 (42%) were positive based on multiplex PCR, and 8 (33%) were positive based on the LightCycler t(14;18) Quantification Kit (mbr) using specific primers for the MBR and JH consensus regions. In total, 15 of 19 patients with grade I FL (79%), 2 with grade II FL (100%), and 4 of the 5 patients with grade III FL (80%) were mbr-positive. In the control group there was no MBR-rearrangement positivity. Among the MBR-negative cases (5/24) none were mcr-positive. Among the 19 patients whose nuclei were isolated, all but 1 were t(14;18) positive based on FISH.

## DISCUSSION

The incidence of the t(14;18) (q32;q21)-IgH/bcl-2rearrangement in FL is 70%-95% (16;17). There is no gold standard technique for detecting t(14;18), and a combination of conventional cytogenetics, southern blot (SB), and PCR techniques is generally used [18]. Initial molecular tests were based on SB techniques, using probes homologous to MBR and mcr sequences. SB hybridization is highly sensitivity, but is labor-intensive and time-consuming, requiring the use of radioactive isotopes and high-quality DNA, which is never obtained from paraffin-embedded biopsy specimens [19]. Recently, PCR and FISH have been used to detect IgH/bcl-2 translocation.

Molecular methods can be negatively affected by such factors as specimen age, initial tissue handling, fixative, and the paraffin-embedding process. PCR and FISH are molecular techniques commonly used in clinical laboratories to detect t(14;18) (q32;q21)-IgH/bcl-2 that lack these limitations. Both tests, however, are prone to technical failure [1]. Fixation is a significant problem for PCR and FISH. When paraffin-embedded specimens are fixed in mercuric chloride-containing fixatives such as B5-formalin, the efficiency of the DNA isolation method decreases. In the present study the DNA and nuclei isolation rate in specimens that were fixed in B5-formalin and embedded in paraffin (n=12) was 42% and 33%, respectively, versus 95% and 60% in the 20 samples fixed in formalin, respectively. 

Paraffin extraction methods typically yield less than fragmented template, and mercuric chloride-containing fixatives can negatively affect DNA integrity and may hamper binding of the primer to the target sequence, often resulting in test failure [20]. In the present study the MBR positivity rate in the DNA of tissue samples was 82.14% based on semi-nested PCR, 53.57% based on multiplex PCR, and 28.57% based on real-time PCR. Among 5 MBR negative samples that were analyzed, mcr rearrangement and mcr positivity were not detected; we think that mcr rearrangement was not observed due to DNA degradation. However we have investigated a new method for mcr rearrangement by using SYBR Green I. and the sensitivity of the semi-nested PCR for MBR rearrangement was detected [79%]. Which is accepted as higly succesfull. 

Similarly, Einerson et al. reported that DNA was successfully amplified in 42% (10/24) of B5 formalin-fixed specimens and that IgH/bcl-2 was detected via multiplex PCR in 5 (36%) of successfully amplified cases [21]. Gisele et al. reported 60% (18/30) MBR positivity in paraffin embedded tissue samples and 63% (19/30) in blood samples [2]. Light Cycler t(14;18) Quantification Kit (MBR) analysis in the present study showed that 8 patients were positive, with scores ranging between 0.00759 and 0.28. As such, semi-nested PCR was superior for the detection of MBR positivity, as compared to the other methods used in the present study. In another study on the frequency of t(14;18) in Turkish FL patients Sayhan et al. used PCR to evaluate representative tissue blocks from 67 FL patients, 12 with diffuse large B-cell lymphoma, and 11 with reactive hyperplasia. They detected t(14;18) in 46 of the 67 FL patients (68.7%) and 25% of those with diffuse large B-cell lymphoma; in the FL cases 64.2% of these break points were at the mbr region and 4.5% were at the mcr region [22].

FISH is more sensitive than PCR because it is capable of detecting breakpoints that lie outside the regions covered by PCR, but the success of FISH can be negatively affected by such factors as specimen age, initial tissue handling, fixative type, and the paraffin embedding process. Interphase FISH has been used to characterize hematological malignancies in bone marrow and blood samples. In particular, with fresh tissue samples interphase FISH is a useful ancillary technology that plays an important role in the differential diagnosis and classification of lymphoma [23]. Several reports of the use of FISH with paraffin-embedded lymphoma biopsy material have been recently published [14]; however, FISH is still not widely used for routine diagnosis, probably because it is perceived to be technically demanding and costly [24]. 

Because of its feasibility, we used FISH method in the present study on isolated individual nuclei from paraffinembedded tissue. Of the 19 specimens whose nuclei were is olated, only 1 patient had a negative result. Sarah et al. studied 5 specimens cytogenetically proven positivity of t(14;18) (q31,q21) for bcl-2 and IgH, in bone marrow from these patients and 1 normal lymph node [12]. They showed that 5 patients had t(14;18) (q31;q21) associated with reciprocal fusion of portions of bcl2 and IgH loci. 

In the present study nested-PCR was used to detect MBR rearrangement, as the technique is capable of detecting just 1 t(14;18)-positive cell from among 10,000 normal cells. 

It is obvious that FISH is a more time-consuming technique than nested PCR. PCR is faster than other molecular methods, can be performed with poor-quality DNA, and has high sensitivity, allowing the use of archival material such as formaldehyde-fixed paraffin-embedded samples. When the results cannot be obtained with PCR due to laboratory conditions and sample quality, FISH can be used. 

Paternoster et al. reported that FISH works equally well as PCR with formalin- or B5-formalin-fixed and paraffin embedded tissue samples; however, in the presence of limitations due to specimen age and fixation methods, FISH has a higher success rate than PCR in [24 of 28 samples with the ratio of 86%] [25]. Many patients with FL respond to treatment, but complete remission rates are very low. For investigated abnormalities, multiplex PCR and nested PCR results were evaluated as positive in 1 patient in the present study, whereas FISH could not show any result in remission; however, the patient relapsed 6 months following this analysis and early positivity of multiplex and nested PCR in this patient was evaluated as the early warning for the clinician in retrospective manner.

Several methods have been developed to detect IgH/ bcl-2 translocated sequences, although no single technique can detect the genetic lesions underlying deregulated bcl-2 expression in all cases of FL [19]. We have concluded that PCR and FISH methods are complementary to each other. The t(14;18)(q32;q21) chromosomal translocation induces bcl-2 protein expression in FL that is identified immunohistochemically using routine pathology diagnostic algorithms; however, a small number of cases lack bcl-2 protein expression, despite carrying the t(14;18)(q32;q21) translocation, due to somatic mutations of the translocated bcl-2 gene, which prevents epitope recognition by bcl-2 antibodies [26]. In such cases FISH and/or PCR analysis may be needed to confirm the pathologist’s diagnosis. Thus, the present study aimed to evaluate the role of these molecular techniques in the diagnosis and follow-up of FL, despite the fact that monitoring of t(14;18) in blood, bone marrow, and lymph nodes in FL patients remains controversial and further studies are needed to clarify this issue. As well known, t(14;18) (q32;q21) translocation may be seen in patients with diffuse large B-cell lymphoma with aggressive presentation, especially those with concurrent 8q24/c-MYC rearrangements; therefore, this translocation is not specific to FL. Because the majority of FL patients with advanced stage disease relapse following diagnosis due to the persistence of residual disease, FISH and/or PCR-detected IgH/bcl-2 rearrangement may be related to the original FL clone developed and detected during follow-up of these patients. Molecular-based studies have shown that t(14;18)-bearing cells may persist in the lymph nodes of FL patients that are in complete remission [27]. Additionally, PCR-based techniques can also detect the MBR/JH fusion sequence in the blood and lymphoid tissues of healthy individuals [28,29]. It has been shown that t(14;18) is generated during early B-cell development in the bone marrow and that affected cells may mature and expand in germinal centers [30]. As such, the most reliable technique for diagnosis and follow-up of FL is histopathological plus immunohistochemical analysis, but FISH—and in some cases PCR— may be helpful in cases with negative bcl-2, CD10, and/or bcl6 immunohistochemistry results. 

In conclusion, we think that using both PCR and FISH can facilitate the genetic characterization of tumors. Detection of specific abnormalities in individual patiens would be helpful to develop patient-specific therapies. 

**Acknowledgements**

This study was supported by the Ege University Medical Research Council (no. 06-TIP-006).

## CONFLICT OF INTEREST STATEMENT

The authors of this paper have no conflicts of interest, including specific financial interests, relationships, and/ or affiliations relevant to the subject matter or materials included.

## Figures and Tables

**Table 1:**
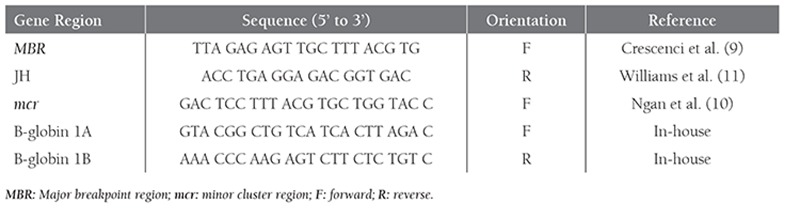
Multiplex PCR primers used for detecting IgH/ bcl-2 rearrangement.

**Table 2:**
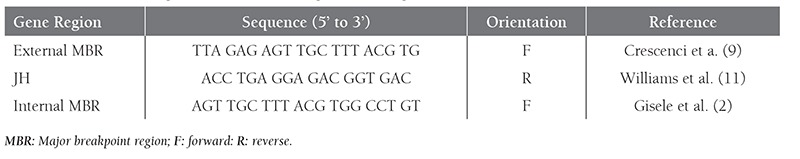
Semi-nested PCR primers used for detecting MBR rearrangement.

**Table 3:**
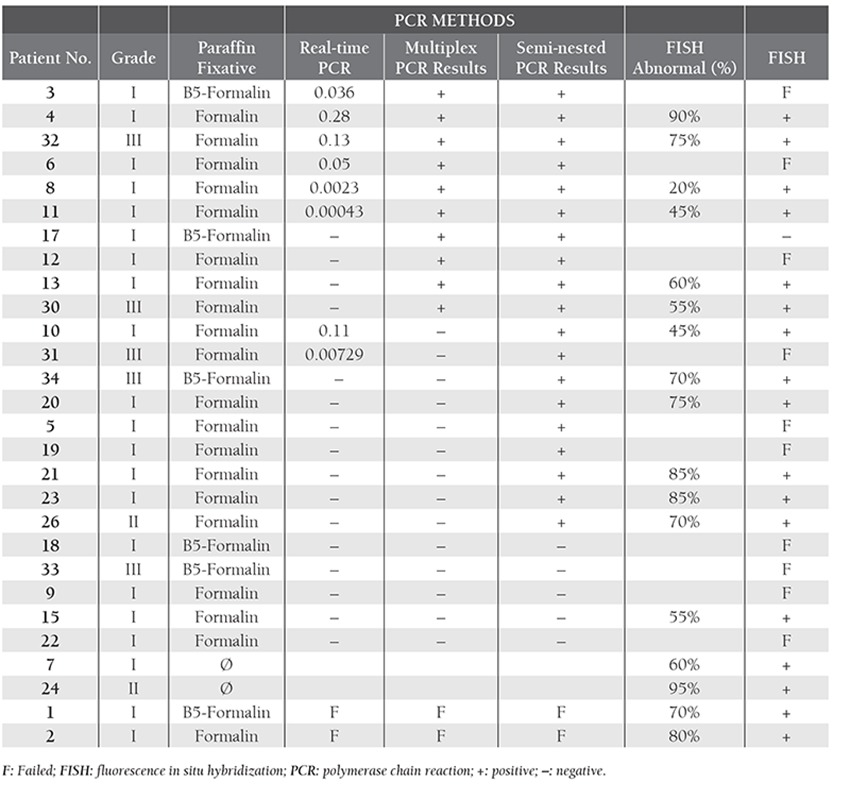
Comparison of PCR and FISH results.
